# Modifiable risk factors for age‐at‐onset of Alzheimer’s disease through genetic determinants

**DOI:** 10.1002/alz.091042

**Published:** 2025-01-09

**Authors:** Yi‐Ju Li, Jong ok La, Qiao Fan, Eden R. Martin

**Affiliations:** ^1^ Duke Molecular Physiology Institute, Duke University School of Medicine, Durham, NC USA; ^2^ Department of Biostatistics and Bioinformatics, Duke University School of Medicine, Durham, NC USA; ^3^ Centre of Quantitative Medicine, Duke‐NUS Medical School, Singapore Singapore; ^4^ Dr. John T. Macdonald Foundation Department of Human Genetics, University of Miami Miller School of Medicine, Miami, FL USA; ^5^ John P. Hussman Institute for Human Genomics, University of Miami Miller School of Medicine, Miami, FL USA

## Abstract

**Background:**

Age and genetic predisposition are well‐known, non‐modifiable risk factors for Alzheimer’s disease (AD). Epidemiological studies have linked several modifiable risk factors to AD, but less is known about their influence on the age‐at‐onset (AAO) of AD. With an increase of genome‐wide association studies (GWASs) and advanced genetic analysis developed, we sought to identify modifiable risk factors for AAO of AD and evaluate their causal effects.

**Method:**

For AAO of AD, we utilized pooled genome‐wide imputed genotype data from 9,219 AD cases and 10,345 controls from 20 cohorts of the Alzheimer Disease Genetics Consortium (ADGC) and our own GWAS summary statistics for AAO of AD (Li et al. 2023). Thirty‐eight exposure factors related to comorbidity, lifestyle, and psychosocial risk factors were screened. The GWAS summary statistics of these exposures were from results released by individual publications or IEU OpenGWAS projects. PRSice‐2 was used to construct polygenic scores (PGS) for each exposure in ADGC subjects. Linear mixed models (LMM) were used to test for association of the exposure‐PGS with AAO, adjusting for sex, APOE‐E4, 10 PCs, and a random intercept by cohort. Exposures meeting p< 0.005 were considered significant risk factors, and they were then evaluated for the causal effect on AAO of AD by a two‐sample Mendelian Randomization (MR) using GWAS summary statistics of exposures (exposure‐GWAS) and AAO of AD. Instrumental variables were selected based on variants meeting p< 5×10^−8^ and p<5×10^−6^, respectively, from the exposure‐GWAS. We evaluated causal effects by the MR inverse variance weighted method and then followed by sensitivity analyses using weighted median and MR‐Egger.

**Result:**

Seven genetically inferred exposures via PGS were significantly associated with AAO of AD (p < 0.005), including higher education attainment and relative fat intake delaying AD onset and type 2 diabetes, cardiovascular disease (CVD), hypertension, and smoking leading to earlier AAO. MR analysis showed lower education, T2D, CVD, and hypertension with significant causal effects on shifting AAO earlier (p<0.007).

**Conclusion:**

The seven modifiable risk factors, particularly, the four causal factors for AAO of AD, will facilitate the early intervention and provide targets to delay AD onset.

